# The arrangements of the microvasculature and surrounding glial cells are linked to blood–brain barrier formation in the cerebral cortex

**DOI:** 10.3389/fnana.2024.1438190

**Published:** 2024-08-07

**Authors:** Yukari Shigemoto-Mogami, Kimiko Nakayama-Kitamura, Kaoru Sato

**Affiliations:** Division of Pharmacology, Laboratory of Neuropharmacology, National Institute of Health Sciences, Kawasaki, Japan

**Keywords:** microglia, astrocytes, BBB, development, three-dimensional distribution, glia limitans

## Abstract

The blood–brain barrier (BBB) blocks harmful substances from entering the brain and dictates the central nervous system (CNS)-specific pharmacokinetics. Recent studies have shown that perivascular astrocytes and microglia also control BBB functions, however, information about the formation of BBB glial architecture remains scarce. We investigated the time course of the formation of BBB glial architecture in the rat brain cerebral cortex using Evans blue (EB) and tissue fixable biotin (Sulfo-NHS Biotin). The extent of the leakage into the brain parenchyma showed that the BBB was not formed at postnatal Day 4 (P4). The BBB gradually strengthened and reached a plateau at P15. We then investigated the changes in the configurations of blood vessels, astrocytes, and microglia with age by 3D image reconstruction of the immunohistochemical data. The endfeet of astrocytes covered the blood vessels, and the coverage rate rapidly increased after birth and reached a plateau at P15. Interestingly, microglia were also in contact with the capillaries, and the coverage rate was highest at P15 and stabilized at P30. It was also clarified that the microglial morphology changed from the amoeboid type to the ramified type, while the areas of the respective contact sites became smaller during P4 and P15. These results suggest that the perivascular glial architecture formation of the rat BBB occurs from P4 to P15 because the paracellular transport and the arrangements of perivascular glial cells at P15 are totally the same as those of P30. In addition, the contact style of perivascular microglia dramatically changed during P4-P15.

## 1 Introduction

The blood–brain barrier (BBB) is a multicellular vascular structure that separates the central nervous system (CNS) from peripheral blood circulation (Rieckmann and Engelhardt, 2003; Zhao et al., 2015; Sweeney et al., 2019). The BBB maintains CNS homeostasis by tightly controlling the passage of molecules and ions, delivering nutrients and oxygen, and protecting neurons from toxic substances (Obermeier et al., 2013; Zhao et al., 2015). The BBB is composed of brain vascular endothelial cells, basement membranes, pericytes and astrocyte endfeet (Serlin et al., 2015; DeStefano et al., 2018). The physiological functions of the BBB, that is, the precise control of macromolecular passage and metabolic supply, are regulated by tight junction proteins (TJs) that are responsible for the adhesion of tight adhesion of vascular endothelial cells, transporters that modulate the orientation of permeable substances, and neurovascular units (NVUs), which are comprised of surrounding cells, such as neurons, pericytes (Abbott et al., 2010; Keaney and Campbell, 2015; Serlin et al., 2015; Zhao et al., 2015).

Several reports have shown that the initiation of BBB development involves communication between the fetal endothelium and neurons (Stewart and Wiley, 1981) and that maturation occurs during the fetal period (Moos and Mollgard, 1993; Keep et al., 1995; Saunders et al., 2000). However, cerebrovascular remodeling has also been reported to occur very actively in the postnatal rat brain (Harb et al., 2013; Walchli et al., 2015; Coelho-Santos and Shih, 2020). Several reports have shown that the BBB rapidly forms and matures after cerebrovascular network construction (Liebner et al., 2011; Engelhardt and Liebner, 2014). Considering the differentiation and maturation periods of perivascular glial cells, BBB integrity may be strengthened by crosstalk with perivascular glial cells within the neurovascular unit during the postnatal period. However, no such study has investigated the perivascular glial architecture formation of BBB during or after angiogenesis.

At present, astrocytes are known to be a component of the neurovascular unit (Iadecola and Nedergaard, 2007; Abbott et al., 2010). Approximately 90% of the abluminal surface of the cerebral microvasculature is ensheathed by astrocytic endfeet, which act as the second barrier and determine various BBB features. It has been reported that rat cortical astrocytes continue to differentiate to the mature type in the postnatal period (Cayre et al., 2009; Chandrasekaran et al., 2016). Recent reports have suggested that astrocytes are involved in increasing BBB integrity (Serlin et al., 2015; Mader and Brimberg, 2019). In support of this, it was reported that the expression level of apuaporin 4 (AQP4) in astrocyte endfeet is low during the perinatal period (until P7) and rapidly increases thereafter (Haj-Yasein et al., 2011; Lunde et al., 2015). Microglia are resident immune cells in the brain that also maintain homeostasis by altering their own active states under physiological and pathological conditions (Kreutzberg, 1996; Kettenmann et al., 2011; Streit et al., 2014). Microglia originate from yolk-sac-derived myeloid progenitors, which invade the CNS at early stages of brain development (Ginhoux et al., 2010). It has been reported that microglia migrate into the brain before developing blood vessels and neural circuits. In the developing brain, microglia have been reported to play many physiological roles, such as removing apoptotic cells, promoting the neurogenesis and gliogenesis, and the pruning of extra spines (Pont-Lezica et al., 2011; Michell-Robinson et al., 2015; Reemst et al., 2016; Tay et al., 2017; Shigemoto-Mogami and Sato, 2021). Microglia have been reported to induce vascular branching during cerebral blood vessel development (Zhao et al., 2015). However, little is known about the development of perivascular microglia and their functions during BBB formation.

In this study, we investigated the perivascular glial architecture formation along with the BBB formation in the rat cerebral cortex. We first checked the integrity of the BBB using Evans blue and tissue fixable biotin, which cannot pass through the mature BBB. Then, we examined the architectures and the attachments to blood vessels of perivascular glial cells via 3D reconstruction image analysis. Our results suggest that the “perivascular glial architecture formation” of BBB is formed between P4 and P15.

## 2 Materials and methods

### 2.1 Animals and treatment

All animals were treated in accordance with the *Guidelines for the Care and Use of Laboratory Animals* published by the National Institute of Health Sciences. All experiments were approved by the Animal Research Committee of the National Institute of Health Sciences and conformed to the relevant regulatory standards. Wistar rats were purchased from Japan SLC and maintained under specific pathogen-free conditions at a controlled temperature and humidity and on a 12 h light/12 h dark cycle with *ad libitum* access to food and water. Ten to twelve rat pups were obtained from each pregnant rat litter, dye penetration tests and immunostaining experiments were performed at each postnatal time point, and comparisons were made between litters. Three to five animals were used at all time points in each experiment.

### 2.2 Determination of BBB formation (tight junction completion) timing in postnatal rats by Evans blue administration

To evaluate BBB formation timing, 2% Evans blue (3 mL/kg) or the same volume of PBS was intraperitoneally injected into rats of either sex once at postnatal days (P) 1, 3, 9, 14, or 29. Twenty-four hours after EB injection, the rats were subjected to cardiac perfusion with saline followed by 4% PFA, after which the brains and tissues were removed. The degree of blue coloration in the brains of rats administered EB was compared. We verified color changes in cortical areas. To quantify EB leakage from blood vessels to the brain, fluorescence levels (λmax = 605 ± 5 nm) in the parenchyma were measured. From each half of the brain, sagittal sections were cut laterally at a thickness of 30 μm. The sections were incubated for 1 h at room temperature in blocking solution (3% normal goat serum, 0.3% Triton X-100 in PBS) and incubated for 1 h at room temperature in solution containing lectin [DL1177, Vector; 1:200] and DAPI [342-07431, Dojindo, 1:1,000]. Nine sagittal sections were prepared from 3 rats per time point (at p4 and p15), and 40–50 images of the cerebral cortical region and EB coloration were obtained by a Nikon A1R-A1 confocal microscope system. The images were 1,024 × 1,024 pixels with ×20 fields of view, and analysis was performed using NIS-Element analysis software. We created 8 ROIs per image and quantified them. The ROI was created with a size of 160 μm^2^ approximately 50 μm from the blood vessel. The mean ROI values were averaged for each image. The data of control animals not receiving EB were subtracted at each time point.

### 2.3 BBB formation (tight junction formation) timing determination in postnatal rats by tissue fixable biotin administration

To evaluate BBB formation timing, an experiment evaluating the leakage of tissue-fixed biotin (Sulfosuccinimidbiotin; Sulfo-NIH Biotin) was conducted with reference to previous research (Daneman et al., 2010). The P1, 4, 10, 15, and 30 rats were transcardially perfused with Sulfo-NIH Biotin (1 mg/mL) for 3–5 min, followed by 5 min of perfusion with 4% PFA at a rate of 0.7 mL/min. Then, the brains were dissected and fixed in 4% PFA overnight at 4°C before being submerged in 30% sucrose. From each half of the brain, sagittal sections were cut at a thickness of 30 μm. The sections were incubated for 3 h at room temperature in blocking solution (3% normal goat serum, 0.3% Triton X-100 in PBS) and incubated for 1 h at room temperature in a solution containing Alexa 546 Streptavidin [S11225, Invitrogen; 1:500]. After washing, the sections were stained with lectin [DL1177, Vector; 1:200] and DAPI [342–07431, Dojindo; 1:1,000]. We used lectin staining to visualize blood vessels instead of the immunostaining of CD31 because the expression level of CD31 depends on the developmental stage (Lossinsky et al., 1997; Lossinsky and Wisniewski, 1998). The stained sections were analyzed using a Nikon A1R confocal microscope system. Sagittal sections were prepared, and the leakage of biotin was quantified using NIS-Element analysis software. Images with a × 20 field of view were obtained, and 6 blood vessels per image were measured. To quantify the biotin leakage fluorescence values, a line was drawn perpendicular to a blood vessel, and the highest biotin value on the vessel was set as 100. The sum of the fluorescence values on the line 20 μm left and right from the blood vessel was calculated and normalized to the highest value on the blood vessel. The fluorescence values of lines in areas without blood vessels were used as the background and were subtracted from the analysis data of the same image. At each time point, 70–76 blood vessel cross sections in 12–14 field images of the cerebral cortex regions obtained from three rats were quantitatively analyzed.

### 2.4 Immunohistochemistry (sagittal sections)

Rats (the P1, P4, P7, P10, P15, and P30) were anaesthetized and then perfused with PBS, followed by 4% PFA, after which the brains were removed. From each half of the brain, sagittal sections were cut at a thickness of 30 μm. The sections were incubated for 3 h at room temperature in a blocking solution (3% normal goat serum, 0.3% Triton X-100 in PBS) and incubated for 24 h at 4°C in the following primary antibodies: rabbit anti-Iba1 antibody [019-9741, Wako; 1:500], chicken anti-GFAP antibody [ab4674, Abcam; 1:400], and rabbit anti-AQP4 antibody [A5971, SIGMA; 1:500]. After incubation, the sections were washed and incubated for 3 h at room temperature with secondary antibodies (anti-rabbit IgG-conjugated Alexa Fluorochrome or anti-chicken IgG-conjugated Alexa Fluorochrome [Invitrogen; 1:1,000]). After incubation, the sections were washed and incubated for 1 h at room temperature in solution containing lectin [DL1177, Vector; 1:200] and DAPI [342-07431, Dojindo; 1:1,000]. After washing, drying, and embedding in VectaShield (H-1000), the stained sections were analyzed using a Nikon A1R-A1 confocal microscope system. Images were taken at intervals of 1.0 μm and 30 μm in the z direction. Images were taken at 1,024 × 1,024 pixels with a 20× field of view.

### 2.5 3D confocal imaging and conformation analysis of glia and blood vessels (Imaris, v.9.5.1)

Z-Stack images of blood vessels (lectin), nuclei (DAPI), and astrocytes (GFAP) or microglia (Iba1) in the rat brain cortical region were acquired with a Nikon A1R-A1 confocal microscope system. Images were taken at intervals of 1.0 μm and 30 μm in the z direction. Images were taken at 1,024 × 1,024 pixels with a 60× field of view. Z-Stack images were opened by Imaris v.9.5.1 and automatically reconstructed into a multichannel 3D image. To clearly understand the shape and distribution of astrocytes, microglia, blood vessels, and nuclei, smoothing surface detail was enabled with a surface grain size of 0.3 μm. To quantify the “astrocyte coverage” or “microglia coverage,” the percentage of vascular tube area covered with AQP4-positive astrocyte endfeet or Iba1-positive microglia, we used Imaris’s XTension algorithm. The XTension algorithm is used to determine the surface contact area between 2 surfaces. The primary surface is the base, and the secondary surface covers the primary surface. XTension generates a one voxel-thick (0.207 μm) unsmoothed surface object above the primary surface, representing where the 2 surfaces physically overlap (white area). This analysis can be used to calculate the total surface area of the formed surface objects. Additionally, the ratio of the new total surface area to the total surface area of a given surface can be calculated.

### 2.6 Statistics

The unpaired *t* test was used for comparisons between p4 and p15 differences in quantified EB fluorescence values. The Scheffe paired comparisons test was used to assess postnatal stage differences in quantified Sulfo-NIH Biotin fluorescence values and cell morphologies (i.e., vessel coverage, contact area, and contact number). Statistical significance was set at *, *p* < 0.05 and **, *p* < 0.01.

## 3 Results

### 3.1 The BBB is formed by postnatal day 10

**Figure 1:**
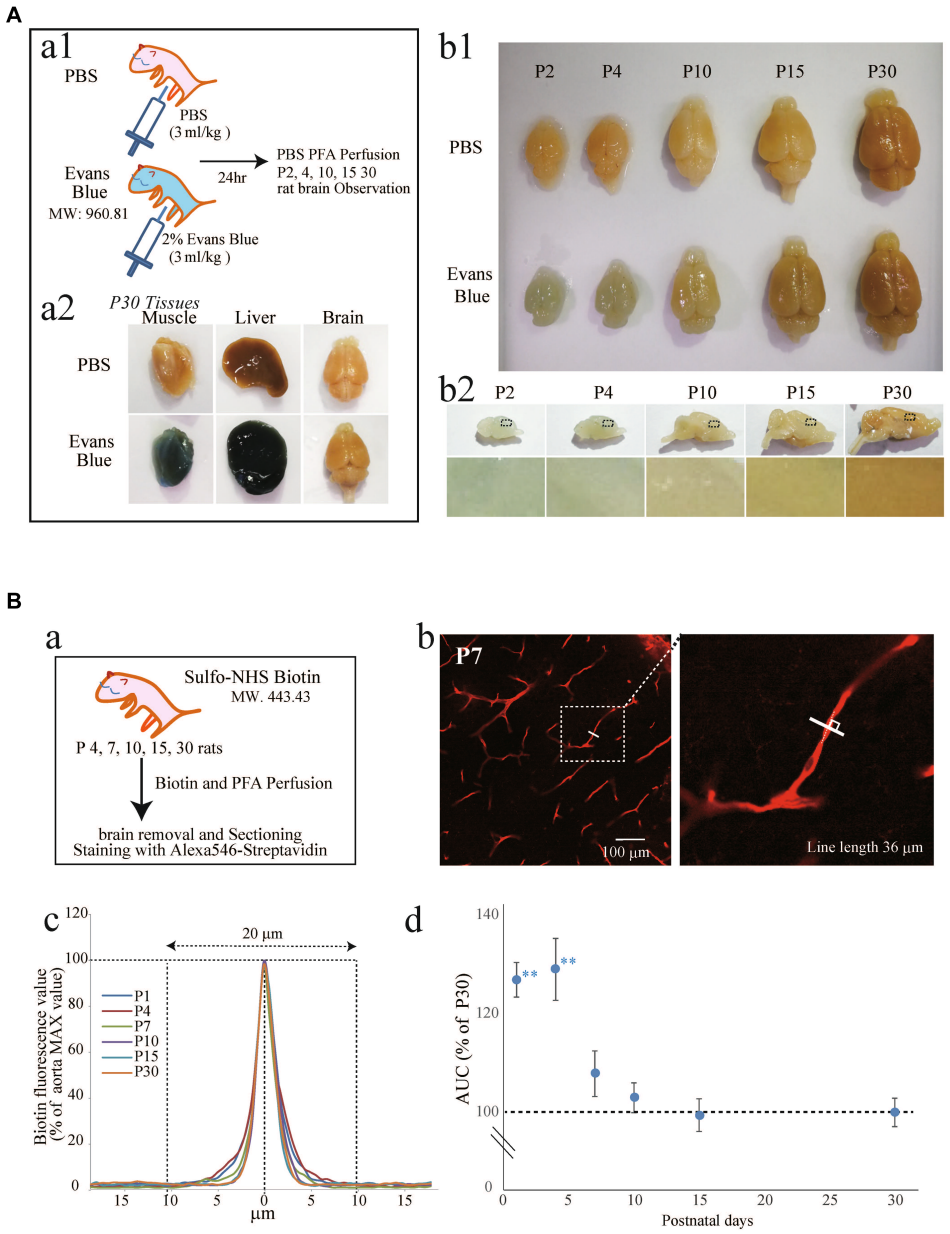
Examination of the BBB formation period in postnatal rats using EB **(A)** and Sulfo-NHS Biotin **(B)**. (**A**, a1) Experimental method diagram. Evans blue was intraperitoneally administered to rats at various stages after birth (the P1, P3, P9, P14, P29). After 1 day, the rats were perfused with 4% PFA for fixation. The brain was removed, and blue coloration was verified. (a2) Brain, liver, and muscle of P30 rats. (b1) Comparison of rat brains at various postnatal periods 1 day after the administration of PBS or EB. (b2): Images showing sagittal brain sections of EB-treated rats. The lower images show the cortical areas of sagittal brain sections from EB-treated rats. The blue color that can be seen in P2 and P4 disappeared as age increased. (**B**, a) Experimental method diagram. Rats at various stages after birth (the P1, P4, P10, P15, and P30) were cardiac-perfused with Sulfo-NHS-Biotin (1 mg/mL) for 3–5 min, followed by 5 min of perfusion with 4% PFA. The brain was removed, and the sections were stained with streptavidin. (b) Shows an image of the P7 rat brain cortical region stained with streptavidin. As shown in the enlarged view, a solid line was drawn perpendicular to the blood vessel indicated by the dashed line, and the red fluorescence value was measured as shown in (c). The traces show the average of 70–76 vessel transverse lines for each postnatal age. The maximum fluorescence values were set as 100, and the sum of the fluorescence values 20 μm to the left and right was calculated. The peak became sharper as the age increased. (d) The graph shows the leakage of the biotin signal in the brain cortical region over time. The vertical axis shows the % of the P30 perivascular streptavidin fluorescence value, which is the streptavidin fluorescence value of the measurement line—the background value. The data are expressed as the mean ± SEM of 70–76 blood vessel cross-sections from 12 to 14 field images of the cerebral cortex regions (635 × 635 μm image) obtained from three rats (4–5 region images were obtained from each rat). **p < 0.01, P1, P4, versus P30, according to Scheffe’s paired comparisons test.

Although it has been reported that the rat cerebrovascular system rapidly develops and functionally matures after birth (Liebner et al., 2011; Engelhardt and Liebner, 2014), the timing of BBB formation remains to be elucidated. Therefore, we first investigated the timing of BBB formation, i.e., tight junction completion, in rat cortices by two established methods (Sun et al., 2021; Wei et al., 2021). We first examined the permeability of the BBB in P1–P30 rats to EB (Figure 1A) and Sulfo-NHS Biotin (Figure 1B). EB and Sulfo-NHS Biotin are commonly used to assess BBB integrity in *in vivo* experiments because they cannot permeate the maturated BBB (Daneman et al., 2010). The experimental scheme is shown in Figures 1Aa1,Ba, respectively. When EB was intraperitoneally administered to P30 rats (Figure 1Aa1), the skin, liver, and hind leg muscle turned blue the next day (Figure 1Aa2), while the color of the brain did not change (similar to that of the PBS-treated control rats). We then injected EB into the P1, P3, P9, P14, and P29 rats and observed the brain the next day (Figure 1Ab1: whole brains; Figure 1Ab1: sagittal cross sections). The rat brains (Figures 1A,B) and extravascular parenchymas in the cerebral cortex (Figure 1Ab2) were colored blue until postnatal Day 4, and this blue color disappeared after postnatal Day 10. EB itself emits fluorescence, so we measured the fluorescence intensity in the cerebral parenchyma of P4 and P10 rats (Supplementary Figure S1). The intensity in P15 rats was significantly lower than that in P4 rats.

Because EB fluorescence is rather weak for quantification, we also used sulfo-NHS Biotin amplified by Alexa 546-streptavidin (Figure 1Ba) (Daneman et al., 2010). The fluorescence intensity on an orthogonal line (80 μm) of a blood vessel (Figure 1Bb, inset), which was positioned in the center of the blood vessel, was quantified. A fluorescence intensity stronger than the baseline (>20 μm of the center, as shown in Figure 1Bc) indicates leakage of the Sulfo-NIH Biotin. Therefore, we quantified the areas between the curves and baselines for the P1, P4, P10, P15, and P30 and normalized them to the P30 value (Figure 1Bd). After P4, the leakage dramatically decreased, and the values for P15 and P30 were almost the same, suggesting that BBB integrity plateaued after P15.

### 3.2 Morphological and configurational changes in perivascular astrocytes after birth

**Figure 2:**
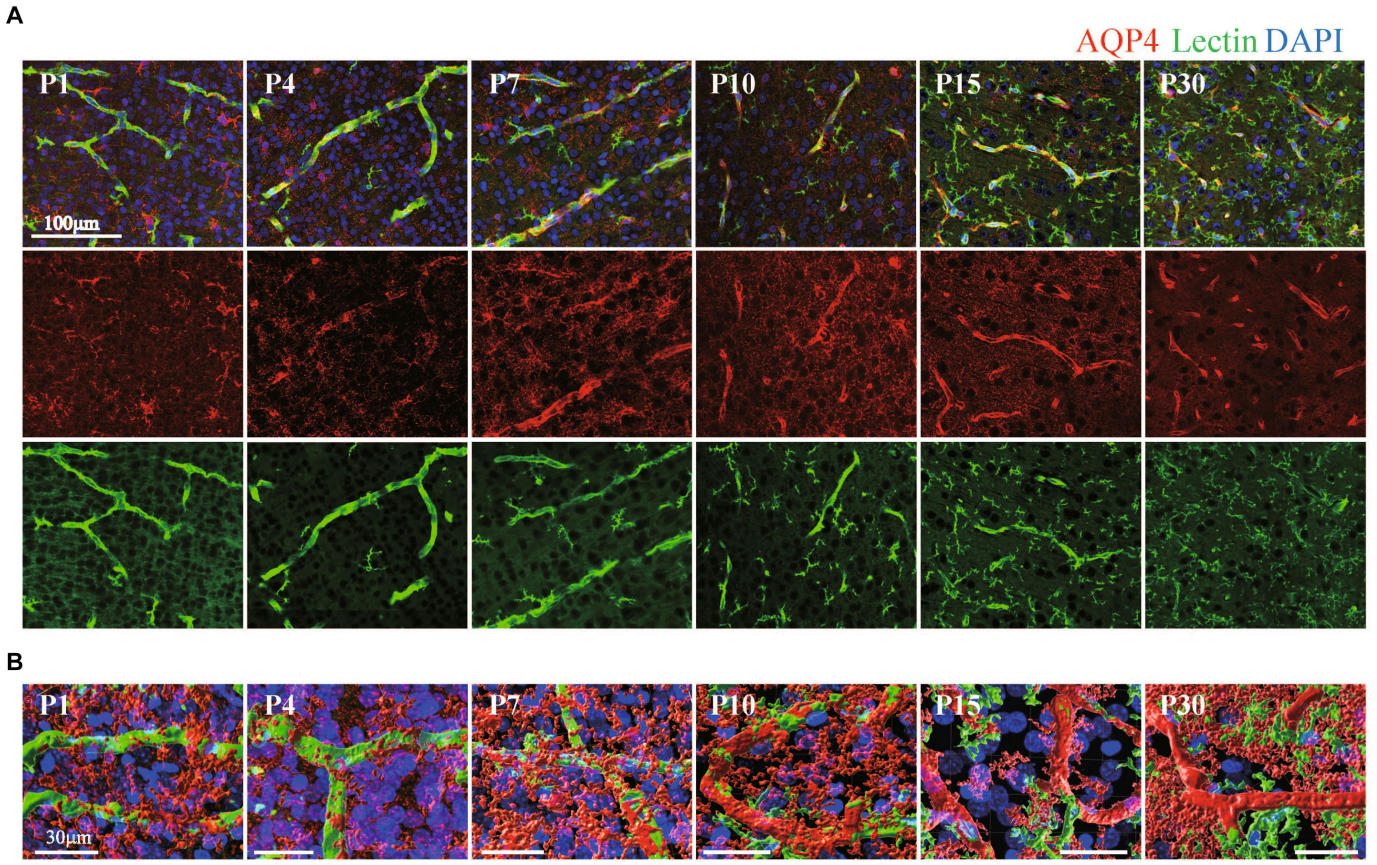
The distribution of AQP4 around blood vessels in the cerebral cortex of postnatal rats. **(A)** Images of AQP4, lectin and DAPI staining in the P1–P30 rat cerebral cortex region are shown. The scale bar indicates 100 μm. AQP4 signals gradually accumulated at blood vessels and were localized to blood vessels after P15. On the other hand, lectin signals were positive in both blood vessels and microglia, and the lectin signals increased after P10 in microglia but not in blood vessels. **(B)** P1–P30 3D images created by Imaris software are shown. The images show AQP4, lectin, and DAPI staining in the cerebral cortex region. The scale bar indicates 30 μm. From P1 to P10, the terminal legs of astrocytes that were positive for the AQP4 signal were in contact with a part of the blood vessel, and the contact area gradually increases. After P15, the vascular tubes were completely covered by the terminal legs of astrocytes.

**Figure 3:**
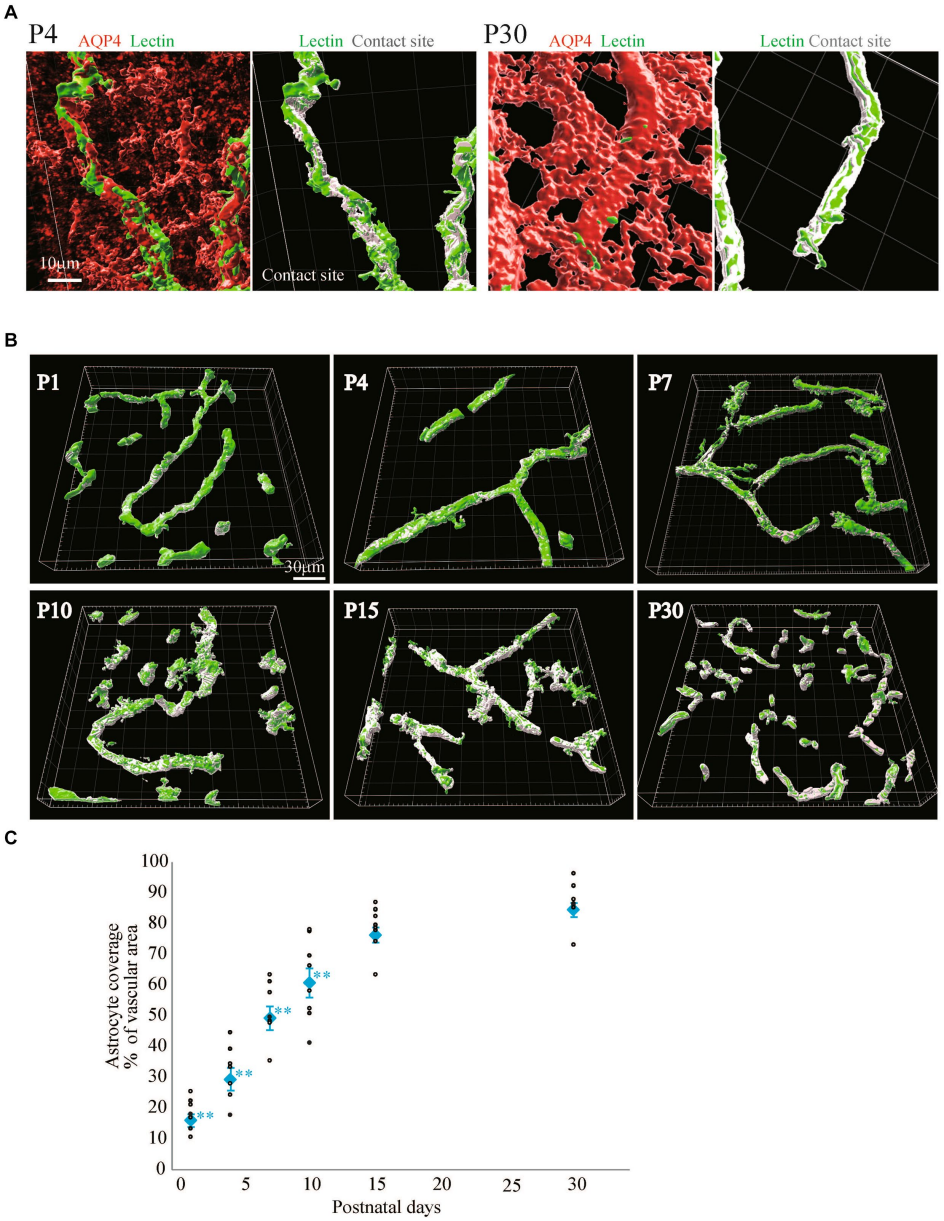
Quantitative analysis of astrocyte coverage at P1–P30. We used Imaris’s XTension algorithm to quantitatively analyze the percentage of vascular tube area covered with AQP4-positive astrocyte endfeets. **(A)** A representative image is shown. The area of the blood vessel covered by the astrocytes is shown in white as the contact site. Most of the blood vessel areas were white at P30 compared to those at P4. **(B)** Representative astrocyte coverage sites on each postnatal day are shown. **(C)** The graph shows the P1-P30 changes in astrocyte coverage (i.e., the proportion of AQP4+ astrocyte endfeet covering cerebral blood vessels). The data are expressed as the mean ± SEM of 7–10 fields of view in the cerebral cortex region 220 × 220 × 30 μm stereoimages obtained from three rats (2–4 region images were obtained from each rats). **p < 0.01, P1, P4, P7, P10 versus P30 by the Scheffe paired comparisons test.

Quite a few reports have mentioned the roles of astrocyte endfeet in BBB functions (Guerit et al., 2021). However, there are few studies showing the morphological changes of perivascular astrocytes along with BBB formation. In this study, we investigated the process by which perivascular astrocytes covered blood vessels by AQP4 immunostaining. AQP4 is a water channel and is generally used to visualize astrocyte morphology. Figure 2 shows the histological signals of AQP4 (red) and lectin (green) in the cortical layer <IV at P1, P4, P7, P10, P15, and P30. From P1 to P30, tubular lectin signals originate from blood vessels (Robertson et al., 2015). At P1 and P4, the AQP4 signals on the lectin+ blood vessels were punctate, suggesting that astrocytes attached to the blood vessels via the heads of their processes. In some astrocytes, AQP4 signals were located throughout the cell body. The AQP+ area on the Lectin+ blood vessels clearly increased with age (Figure 2A). To visualize the configurations of blood vessels and astrocytes more clearly, we re-constructed 3D images from the immunohistochemical data of AQP4+ astrocyte endfeet and blood vessels (Figure 2B). These 3D images verified that the astrocyte-covered areas on the blood vessels became increasingly larger with age. At P15 and P30, astrocytes covered almost all of the blood vessels. Therefore, we changed the color of the AQP4+ astrocyte-blood vessel contact sites and visualized the blood vessels covered by astrocyte endfeet. Using these images, we quantified the “astrocyte coverage” rates of blood vessels using the XTension algorithm in Imaris-installed software, which finds the contact area between two surfaces (Figure 3). Representative XTension-processed images of P4 and P30 are shown in Figure 3A, which reveals the interfaces between AQP+ astrocyte endfeet and blood vessels as white signals. Figure 3B shows the XTension-processed images of the P1, 4, 7, 10, 15, and 30 rats. The white areas gradually increased with age. At P15 and later, white signals covered almost all of the blood vessels. When the white area was normalized to the vascular area in each image, the astrocyte coverage rate started to increase at the P1. At P15 and P30, almost all of the blood vessel surface was covered with AQP+ astrocyte endfeet (Figures 3B,C).

### 3.3 Morphological and configurational changes in perivascular microglia after birth

**Figure 4:**
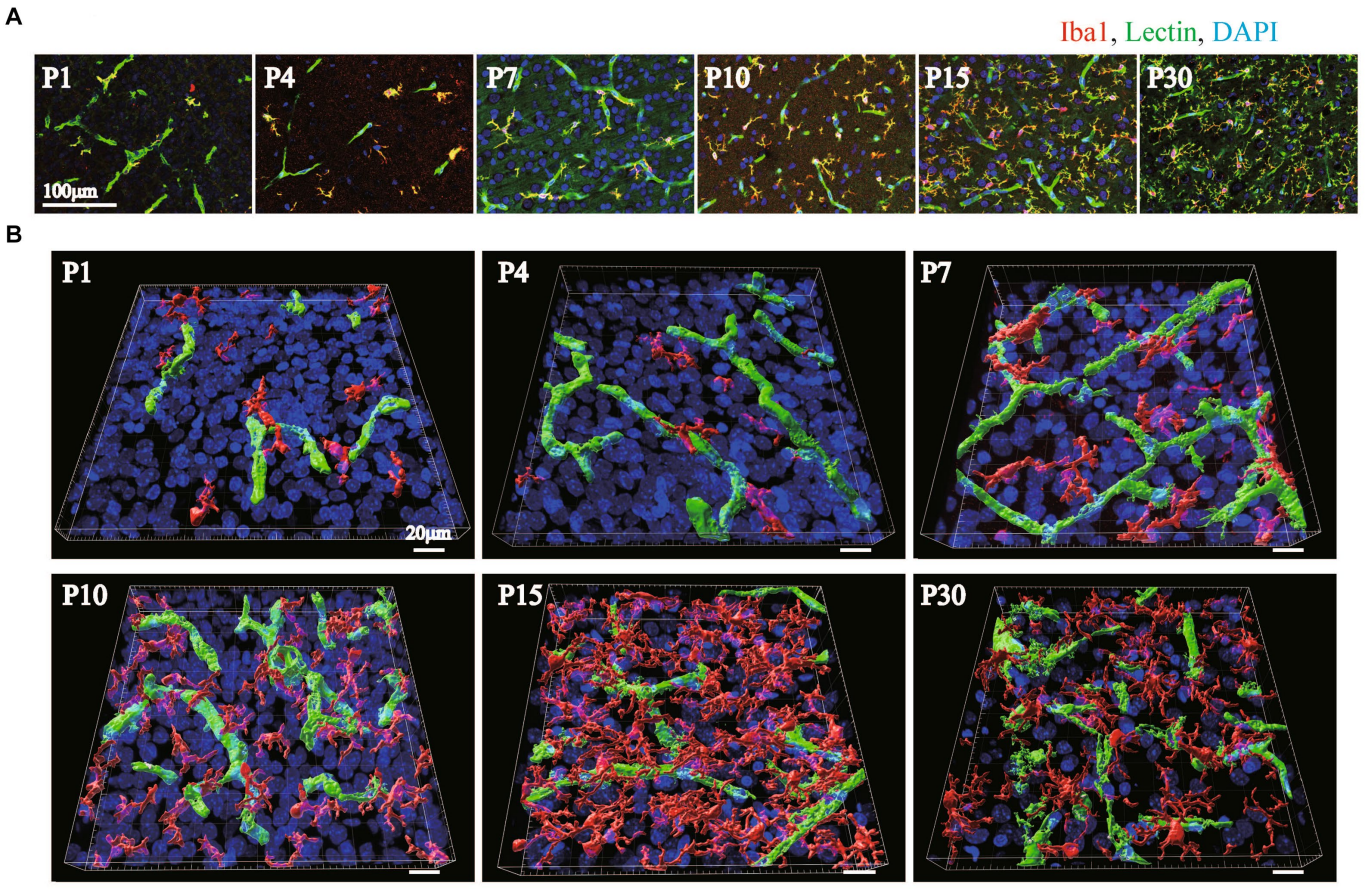
The distribution of Iba1 around blood vessels in the cerebral cortex of postnatal rats (P1–P30). **(A)** Images of Iba1, lectin and DAPI staining in the P1–P30 rat cerebral cortex region are shown. The scale bar indicates 100 μm. Microglia at P1–P30 are Iba1-positive and lectin-positive cells (**A**, yellow cells). As the number of postnatal days increased, the number of microglia in contact with blood vessels gradually increased, and the cell morphology changed to a ramified type with many protrusions. **(B)** P1–P30 3D images created by Imaris software are shown. The images show Iba1, lectin, and DAPI staining in the cerebral cortex region. We created the surface of only blood vessels by removing the costained areas of blood vessels and microglia and investigated the distribution of blood vessels and microglia. The scale bar indicates 30 μm. At P1 and P4, a small number of microglia are in contact with the blood vessels; however, at P7 and P10, the number of microglia gradually increases, and at P15 and P30, the microglia are evenly distributed and contact the vascular network. The number of microglia and the number of contact points were greatest at P15.

**Figure 5:**
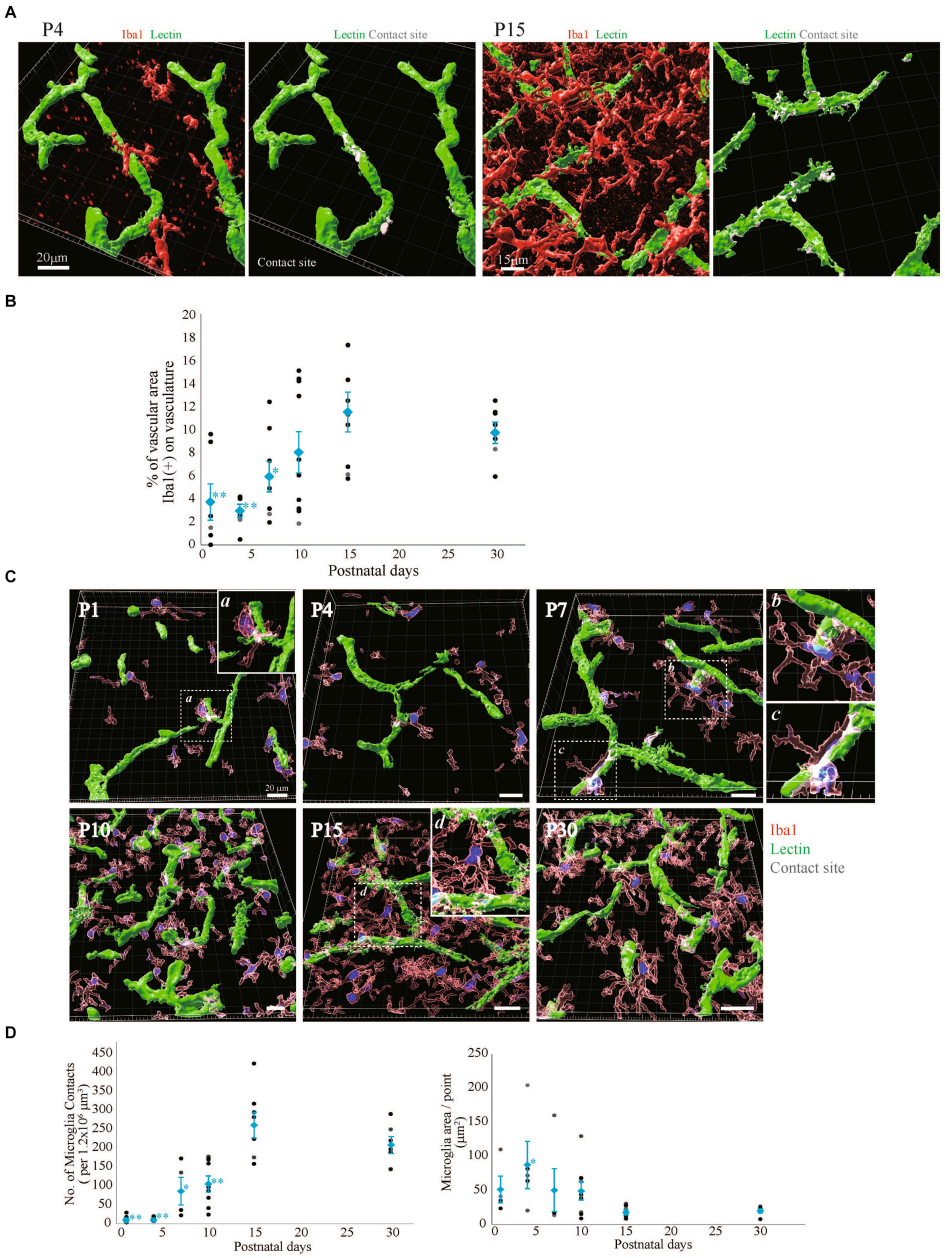
Quantitative analysis of microglial coverage from P1 to P30. We used Imaris’s XTension algorithm to quantitatively analyze the percentage of vascular tube area covered with Iba1-positive microglia. **(A)** Representative images at P4 and P15 are shown. The area of the blood vessel covered by the microglia is shown in white as the contact site. **(B)** Changes in microglial coverage (i.e., the proportion of Iba1+ microglia covering cerebral blood vessels) at P1–P30. The data are expressed as the mean ± SEM of 7–10 fields of view in the cerebral cortex regions of 220 × 220 × 30 μm stereoimages obtained from three rats (2–4 region images were obtained from each rat). **p < 0.01, P1, P4, versus P30, *p < 0.05, P7 versus P30 by Scheffe’s paired comparisons test. **(C,D)** Show the morphology of microglia in contact with blood vessels at P1–P30. **(C)** Representative images at each time point are shown. Microglia are shown in translucent red, microglial nuclei are shown in blue, blood vessels are shown in green, and contact areas between blood vessels and microglia are shown in white. From P1 to P7, microglia were in contact with blood vessels through most of the cell body. a, b, and c are enlarged views and show that the distances between the microglial nucleus and blood vessels were also very close. At P7, various microglial shapes were observed. After P10, the cell bodies of microglia are located far from blood vessels, and microglia make contact with blood vessels through their protrusions. d is an enlarged view of microglia contacting two blood vessels with their protrusions. **(D)** A graph of the number of microglial contacts and the average contact area of microglia within the stereo image (212 × 212 × 30 μm) is shown. Each dot represents the value in each field of view, and blue dots represent the mean plus SEM. The number of contacts increases rapidly after birth, with the same number of contacts between P15 and P30. The average contact area of microglia reached its maximum at P4 and then significantly decreased. These data indicated that the morphological changes in microglia in contact with blood vessels can be quantified.

It has been reported that microglia dramatically increase in number and change in morphology after birth (Dalmau et al., 1997; Graeber and Streit, 2010; Kettenmann et al., 2011; Cunningham et al., 2013; Shigemoto-Mogami et al., 2014; Michell-Robinson et al., 2015). It has been suggested that microglia are involved in the neurovascular unit and are necessary for BBB functions (da Fonseca et al., 2014; Engelhardt and Liebner, 2014; Dudvarski Stankovic et al., 2016; Haruwaka et al., 2019). In our recent report, we also reported that the interactions of microglia with NVU cells are related to changes in BBB functions under inflammatory conditions (Shigemoto-Mogami et al., 2018). However, little information is available about the roles of microglia in BBB formation. Furthermore, there are no studies showing the distribution and morphological changes of microglia around blood vessels during BBB formation. Therefore, we first investigated the morphologies of perivascular microglia and their structural organization with blood vessels in the P1, P4, P7, P10, P15, and P30 rat cortical layers <IV. We compared the signal intensity of Iba1, a microglial marker, and lectin. In addition to blood vessels, Tomato lectin also stains microglia. We confirmed that Lectin+ signals other than tubular lectin signals were located in Iba1+ cells. The number of Iba1+ microglia rapidly increased until P15, while the morphology dramatically changed to the branched ramified type after P10 (Figure 4A). To clarify the arrangement of the blood vessels and microglia and the morphology of the microglia, we re-constructed 3D images as in the astrocyte study described above (Figure 4B). The number of microglia was greatest at P15. The morphology became a clear ramified type with small cell bodies and multiple processes at P15 (Figures 4A,B). Although microglia contacted blood vessels at all time points, the contact style changed dramatically with age. In terms of the contact of microglia and blood vessels, the microglial cell bodies directly contacted the blood vessels until P7 (Figure 4B). After P10, protrusions from the cell bodies became clearer, and the number of processes increased. As the morphology of the microglia changed, the microglia appeared to start contacting the blood vessels in addition to contacting their cell bodies. To visualize the attachment more clearly, 3D images were re-constructed from immunohistochemical images in a manner similar to that used for visualizing astrocytes (Figure 5). Representative images of P4 and P15 are shown in Figure 5A, in which the interface between Iba1+ microglia and blood cells is white. In the P4 brain, microglia attach to blood vessels through their cell bodies. In the P15 brain, the number of microglia and their processes increased dramatically. Many of the processes contacted the blood vessels, as shown by the white signals. Based on these imaging data, we calculated Iba1+ coverage rate of blood vessels (Figure 5B). The microglial coverage rate increased to 8.1 ± 1.9% until P10, after which it no longer increased. There was no significant difference between P10 and P30 (Figure 5B). Because the attachment style of microglia to blood vessels changed dramatically from P1 to P30, we characterized these changes in more detail. As shown in Figure 5C, at P1 and P4, the number of microglia was still small, and the morphology was ameboid-shaped. They attached to blood vessels with cell bodies (Figure 5Ca). At P7, although ameboid-type microglia were still observed (Figure 5Cc), the morphology of some of the microglia changed to the ramified type, as shown in Figure 5Cb. These ramified microglia contacted blood vessels through their processes. The distances between the blood vessels and their cell bodies (the positions of nuclei) were still close at P7 (Figure 5Cb). After P10, the number of ramified microglia increased, and the contact sites of the processes with blood vessels were confirmed (Figure 5Cd). The attachment of cell bodies and blood vessels was no longer observed. Figure 5Da shows the change in the number of contact points of microglia and blood vessels at the respective ages, while Figure 5Db shows the change in the average area per contact site at the respective ages. The number of microglial contacts increased rapidly by P10, while the average area was widest at P4 and decreased thereafter.

### 3.4 Comparison of microglial attachment to blood vessels with BBB integrity and astrocyte coverage

**Figure 6:**
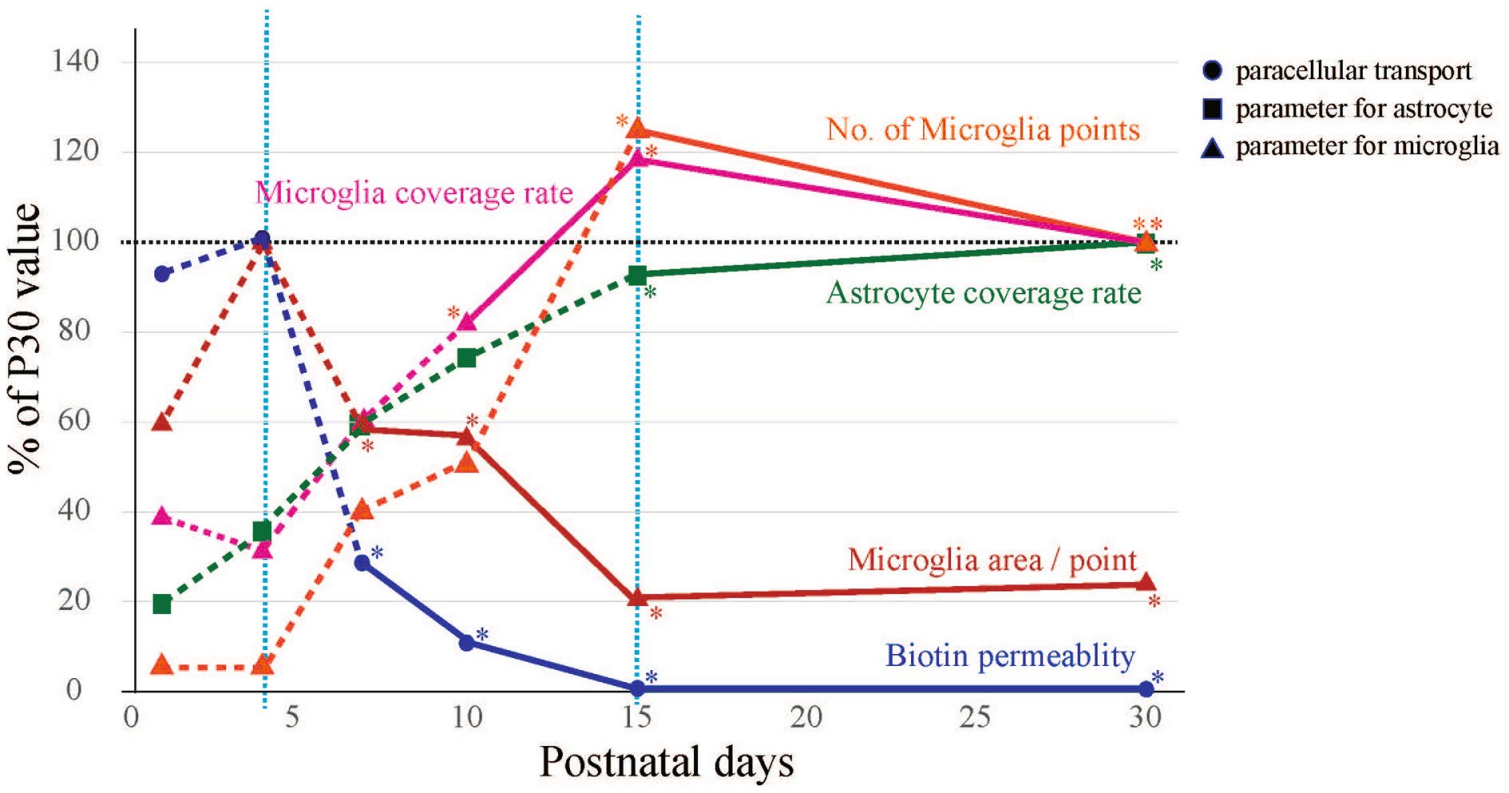
Summary of morphological changes in blood vessels and glia during BBB development. The time-series changes in biotin permeability and microglia area/point were corrected to a maximum value of 100%, and astrocyte coverage rate, microglia coverage rate, and No. of microglia points were corrected to a P30 value of 100%. BBB formation and maturation in rats was classified into three phases from the viewpoints of biotin leakage and astrocyte and microglial contact with blood vessels. The phases are the “immature phase” when biotin permeability is still high, the “developing phase” when glial structures around blood vessels are formed, and the “completion phase” when the structures around blood vessels become stable and the morphology of microglia changes to a ramified type.

We collected the following three kinds of data concerning BBB formation in the cerebral cortex of postnatal rats: (1) leakage of peripherally administered dye into the brain, (2) changes in the attachment of astrocytes to blood vessels, and (3) changes in the attachment of microglia to blood vessels. We integrated these data into one graph (Figure 6), in which biotin permeability and microglial area/point were normalized to the maximum values, while the astrocyte coverage rate, microglial coverage rate, and number of microglial attachments were normalized to the P30 value. This graph shows three phases of perivascular glial architecture formation: “immature phase” (<P4), “developing phase” (P4–P15), and “completion phase” (>P15). We also found that during the “developing phase,” the number and morphology of microglia and their attachment style to blood vessels dramatically changed. These results strongly suggest that in addition to astrocytes, microglia also contribute to BBB formation.

## 4 Discussion

In this study, we succeeded in identifying the BBB formation, i.e., tight junction completion (P4–P15) based on the leakage of EB and Sulfo-NHS Biotin. BBB formation in the cerebral cortex is thought to continue during the postnatal period; however, the perivascular glial architecture formation of BBB during or after angiogenesis remains to be elucidated. We found that there are three phases of perivascular glial architecture formation: “immature phase” (<P4), “developing phase” (P4–P15), and “completion phase” (>P15).

### 4.1 “Immature phase” (<P4)

During this phase, peripherally administered EB and biotin still penetrated the brain parenchyma, suggesting that the BBB intercellular junctions were still insufficient. EB (Manaenko et al., 2011) and biotin (Daneman et al., 2010; Greene et al., 2022) are reliable tools for examining BBB permeability. Previous reports have shown that BBB formation begins at embryonic stages 10–17 through the expression of TJ proteins in cerebral blood vessels (Utsumi et al., 2000; Wolburg and Lippoldt, 2002). TJs become more rigid in brain capillaries after birth (Utsumi et al., 2000), and astrocytes play an important role in TJ formation (Gilbert et al., 2019; Morales et al., 2022). We also confirmed that astrocytes were present around blood vessels in the cerebral cortex at this stage. To visualize the morphology of astrocytes, we used immunohistochemistry to detect AQP4. Although we also immunostained for GFAP, a conventional marker of astrocytes, the strength of the GFAP signal was still weak at this developmental stage. We confirmed that the GFAP signals were colocalized with AQP4 signals in blood vessels (P30). In the case of microglia, we observed that a few ameboid microglia adhered to blood vessels. The number of microglia in the cerebral cortex was still small, and the microglia were ameboid-shaped with few processes. Mondo E et al. reported that microglia at this postnatal age are distributed throughout the brain (Mondo et al., 2020). They are so motile that they migrate along blood vessels and are located throughout the brain (Mondo et al., 2020).

### 4.2 “Developing phase” (P4–P15)

During this period, the permeability of peripherally administered dyes to the brain decreases rapidly and dramatically. The accumulation of AQP4 signals on blood vessels gradually increased; however, even at P15, some parts of the blood vessels were not covered with AQP4+ astrocyte endfeet. These results suggest that astrocyte coverage and barrier functions for dye penetration are independent. This change in the distribution of AQP4 signals is consistent with previous reports (Haj-Yasein et al., 2011; Lunde et al., 2015; Mondo et al., 2020). The colocalization of AQP4 with blood vessels gradually increases after birth (Haj-Yasein et al., 2011; Mondo et al., 2020), and AQP4+ astrocyte endfeet rapidly increase after P7 (Lunde et al., 2015). Microglial coverage and the number of contact points increased rapidly during this period. In addition, the morphology of the microglia changed from the ameboid type to the ramified type, and the processes of microglia contacted the blood vessels, which was the reason why the number of contact points increased while the area per point decreased. These dynamic changes in the attachment style of microglia to blood vessels also suggest that perivascular microglia play important roles in BBB formation. Interestingly, microglial contact points were observed in areas not covered with AQP4+ astrocyte endfeet.

### 4.3 “Completion phase” (P15–)

The parameters for the BBB and glial cells employed in this study were almost the same at P15 and P30. During this period, peripherally administered dyes no longer penetrated the brain. The AQP4+ astrocyte endfeet covered most of the blood vessels. Previous reports suggest that in the adult brain, almost 90 % of the abluminal surface of the cerebral microvasculature endothelium is ensheathed by astrocytic endfeet, which play an essential role in determining various BBB features, in addition to acting as a second barrier (Agrawal et al., 2006). Astrocytes increase TJ expression levels, modulate the distribution of transporters and receptors, and activate enzyme systems (Correale and Villa, 2009; Abbott et al., 2010; Liebner et al., 2011; Engelhardt and Liebner, 2014; Sweeney et al., 2019). Our results indicate that BBB functions induced by the attachment of astrocyte endfeet start to mature after P15. At P15, the microglial coverage rate and the number of microglial contacts reached their maximum, while the microglial contact area per contact site reached its minimum. These morphological changes are consistent with the maturation processes of microglia, which eventually become surveillance ramified microglia (Guedes et al., 2022). Based on these data, we concluded that P15- is the “completion phase.” In the cerebral cortex, however, the net number of neurons at P15 is almost half the number of neurons found in adulthood (Bandeira et al., 2009). Therefore, functional maturation of the BBB, including region-specific increases in functional proteins, may occur after P15.

Generally, astrocytes are the most abundant glial cells in the cerebral cortex. Astrocytes differentiate and mature in the rat cortical region after birth (Cayre et al., 2009; Chandrasekaran et al., 2016). Recent reports have suggested that astrocytes are involved in neurovascular coupling (Iadecola and Nedergaard, 2007; Abbott et al., 2010); therefore, perivascular astrocytes may also differentiate to support the formation and maturation of the BBB. Recent reviews suggest that astrocytes are involved in maturation and maintenance rather than induction of the BBB (Serlin et al., 2015; Mader and Brimberg, 2019). In this study, we focused on the expression of AQP4, which is highly expressed in astrocytic endfeet connected to blood vessels (DeStefano et al., 2018). AQP4 promotes perivascular clearance via the glymphatic system and controls cerebral blood flow (CBF) (Plog and Nedergaard, 2018; Mader and Brimberg, 2019). Previous reports showed that AQP4 covered blood vessels after approximately P14 (Haj-Yasein et al., 2011; Mondo et al., 2020), which is consistent with our results; however, AQP4 is not involved in large molecule permeation.

Microglia are resident immune cells in the brain that maintain homeostasis. Microglia are morphologically classified into two phenotypes: the ramified type and the ameboid type. The ramified-type microglia have branched processes and small cell bodies, while the ameboid-type microglia have large round cell bodies and short processes. In the normal brain, microglia exist in a ramified form and monitor environmental changes within the brain. They also spread out their projections, similar to antennae, and control the generation of neural synapses and synaptic transmission (Nimmerjahn et al., 2005). When abnormalities in the brain are detected by microglia, their shape changes to the ameboid type. Ameboid microglia are proliferative, migratory, and phagocytic. They also release cytokines, chemokines, and growth factors, thereby affecting brain development (Hanisch, 2002; Kettenmann, 2006; Shigemoto-Mogami and Sato, 2021). It has been reported that the morphology and distribution of microglia change dynamically in the rodent brain as the brain develops (Reemst et al., 2016; Van Ryzin et al., 2018). Our data revealed that there were perivascular microglia at the postnatal developmental stage, and the number, shape, and attachment style of these microglia changed dramatically with development. The microglia eventually became ramified. Although recent reports have suggested that perivascular microglia are constituents of the NVU, their interactions with the BBB are largely unknown. Microglial cerebral colonization precedes endothelial cell sprouting. Confocal microscopy and electron microscopy revealed that microglia are closely physically associated with microvessels in both the developing and adult brain (Mondo et al., 2020). When the number of microglia is decreased by PU.1 knockout, vascular network formation is suppressed in the embryonic CNS (Arnold and Betsholtz, 2013). In this study, we clarified the developmental changes in the contact style of microglia with blood vessels using the following parameters: the number of microglial contact points, microglial coverage, and the contact area per point. Interestingly, the contact points of microglia were also observed in areas not covered with AQP4. This finding is consistent with a previous report showing that the AQP4+ signal was low where microglia were in contact with cerebral blood vessels (Mondo et al., 2020). In addition to the interaction between blood vessels and perivascular glial cells, more studies are needed on the interaction between perivascular microglia and astrocytes.

Here we clarified perivascular glial architecture formation along with BBB development. As a next step, we will attempt to link the morphologies of astrocytes and microglia to their roles in macromolecular passage and metabolic supply. It is expected that the attachment patterns of astrocytes and microglia would become benchmarks for determining the BBB formation stage.

## Data availability statement

The raw data supporting the conclusions of this article will be made available by the authors, without undue reservation.

## Ethics statement

The animal study was approved by The Guidelines for the Care and Use of Laboratory Animals published by the National Institute of Health Sciences. All experiments were approved by the Animal Research Committee of the National Institute of Health Sciences and conformed to the relevant regulatory standards. The study was conducted in accordance with the local legislation and institutional requirements.

## Author contributions

YS-M: Conceptualization, Data curation, Formal analysis, Investigation, Methodology, Project administration, Visualization, Writing – original draft. KN-K: Data curation, Investigation, Visualization, Writing – original draft, Validation. KS: Data curation, Investigation, Validation, Visualization, Writing – original draft, Conceptualization, Formal analysis, Funding acquisition, Methodology, Project administration, Resources, Supervision, Writing – review & editing.
