# Supplementary figures and images for: The arrangements of the microvasculature and surrounding glial cells are linked to blood–brain barrier formation in the cerebral cortex
Source: Front Neuroanat. 2024 Aug 7;18:1438190. doi: 10.3389/fnana.2024.1438190 (PMC11335649; doi:10.3389/fnana.2024.1438190)

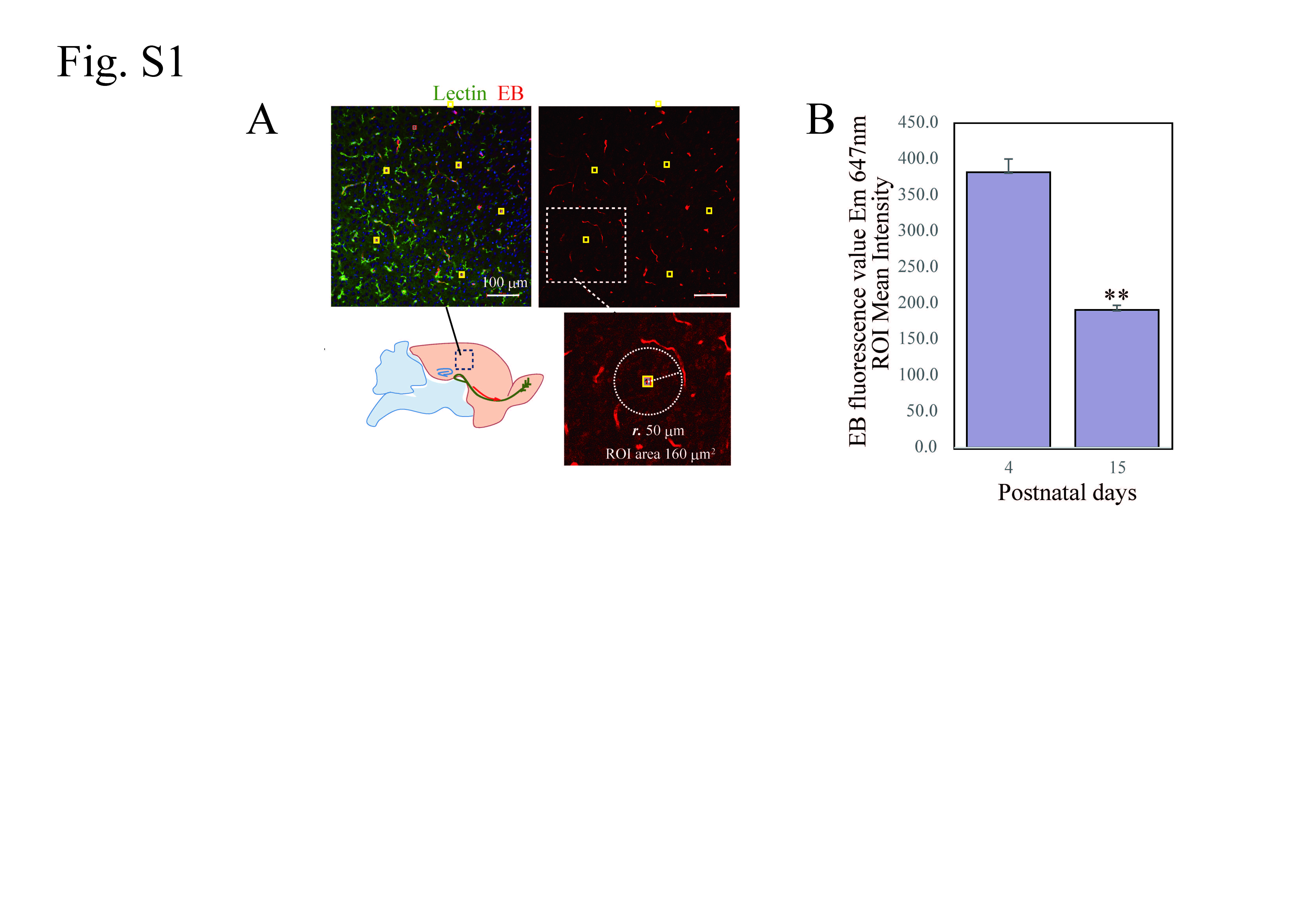

Supplement: Supplementary file 1 [file Image_1.JPEG]
